# Novel Link Between Myeloid-Specific Adenosine Deaminase 2 and CXCL10-CXCR3 Axis in Infectious ARDS

**DOI:** 10.3390/ijms26083678

**Published:** 2025-04-13

**Authors:** Shilpa Tiwari-Heckler, Yered Pita-Juarez, Lisa Vierbaum, Patrick Michl, Ioannis S. Vlachos, Uta Merle, Z Gordon Jiang

**Affiliations:** 1 Department of Gastroenterology, Infectiology and Toxicology, University Hospital Heidelberg, 69120 Heidelberg, Germany; 2 Department of Pathology, Beth Israel Deaconess Medical Center, Harvard Medical School, Boston, MA 02215, USA; 3 Department of Medicine, Division of Gastroenterology, Beth Israel Deaconess Medical Center, Harvard Medical School, Boston, MA 02215, USA

**Keywords:** infiltrative monocytes, ARDS, COVID-19, purinergic signaling

## Abstract

Acute respiratory distress syndrome (ARDS) is a severe complication of lung injury characterized by hyperinflammation and fibrosis. Here, we show a significant association between the monocyte-derived enzyme adenosine deaminase 2 (ADA2) and SARS-CoV-2 induced ARDS. We note an interesting link between ADA2 and the chemokine CXCL10 and its receptor CXCR3. By using published datasets of spatial transcriptomics and single-cell RNAseq, we show that ADA2 is highly expressed by inflammatory CD14^+^CD16^+^ monocytes, along with profibrotic genes, in lungs affected by COVID-19. This study reveals important associations between key pathophysiological features of ARDS, linking hypoxia, infiltrative CXCR3 monocytes, and a monocyte-derived exoenzyme ADA2.

## 1. Introduction

Acute respiratory distress syndrome (ARDS) is caused by various pathogens of bacterial and viral origin, such as severe acute respiratory coronavirus type 2 (SARS-CoV-2). The resulting coronavirus disease (COVID-19) led to a global pandemic with high mortalities, largely due to severe complications such as ARDS. Despite significant research efforts, there are currently no effective treatments to improve patient outcomes for infectious ARDS [1].

Mounting evidence suggests that ARDS is not only mediated by virus-induced mucosal damage but also driven by dysregulated innate immune responses. Monocytes and macrophages play critical roles in first-line host defense and inflammation, as well as in tissue repair and fibrosis [2]. Impaired responses of these cells to SARS-CoV-2 are believed to be the main cause of hyperinflammation and pulmonary fibrosis in SARS-CoV-2 induced ARDS [1,2]. We recently reported that infiltrative monocyte-derived macrophages with strong expression of adenosine deaminase 2 (ADA2) modulate liver inflammation and fibrosis [3]. Since purinergic pathways involving ATP- and adenosine-mediated signaling have been noted to be critical in modulating lung injuries [4], we aimed to investigate whether this novel macrophage-specific enzyme, ADA2, could be involved in the pathogenesis of SARS-CoV-2-induced ARDS.

## 2. Results

We retrospectively analyzed blood cells and serum samples derived from patients with COVID-19-associated ARDS and those with mild-to-moderate COVID-19 symptoms who did not develop ARDS (non-ARDS group). Patients were assigned to the ARDS group within seven days of worsening respiratory symptoms and intubation in accordance with the Berlin criteria, as well as a PaO_2_/FiO_2_ < 200 [5]. Clinical characteristics of both groups are summarized in Table 1.

**Figure 1:**
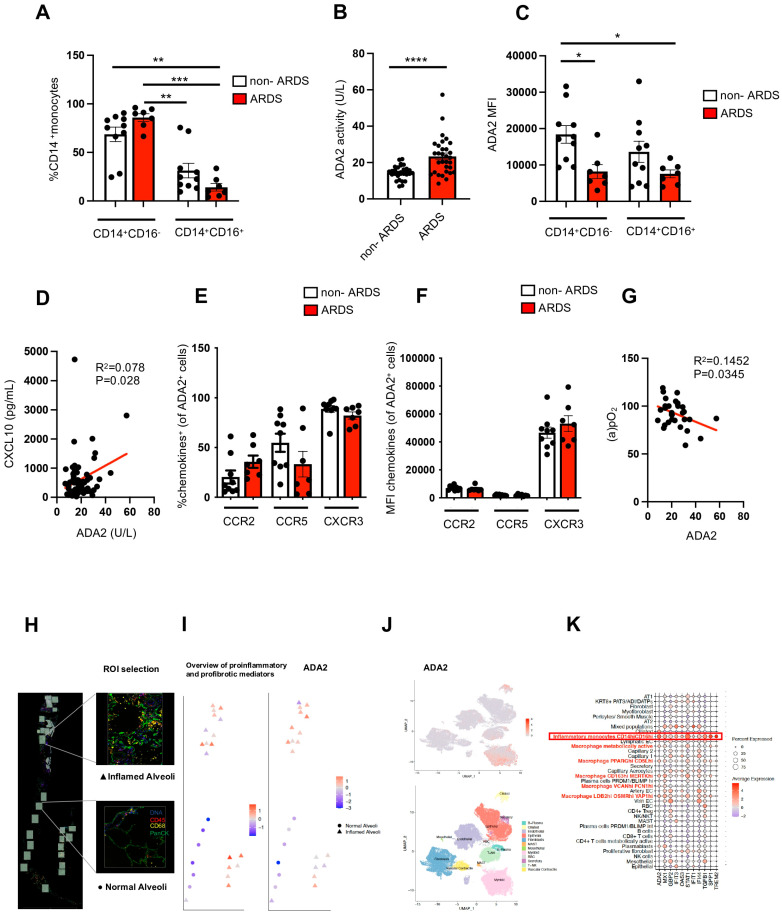
Monocyte-derived adenosine deaminase 2 is associated with SARS-CoV-2-induced ARDS. (**A**) Frequency of CD16^−^ and CD16^+^ cells in CD14^+^ monocyte cell population in circulation of non-ARDS (n = 10) and ARDS groups (n = 7). (**B**) Serum ADA2 activity in non-ARDS (n = 33) and ARDS groups (n = 34). (**C**) Mean fluorescence intensity (MFI) of ADA2 measured in CD14^+^CD16^−^ and CD14^+^CD16^+^ cell population in non-ARDS (n = 10) and ARDS groups (n = 7). (**D**) Linear regression analysis of serum level of CXCL10 and ADA2 activity in non-ARDS and ARDS groups. (**E**) Frequency and (**F**) MFI of chemokine receptors CCR2, CCR5 and CXCR3 in blood-derived ADA2^+^ cells in non-ARDS (n = 10) and ARDS groups (n = 7). (**G**) Linear regression analysis of serum ADA2 activity and partial pressure of arterial oxygen (a)pO_2_). All bar figures represent mean ± standard error of the mean. *p* value obtained using Mann–Whitney U test or Kruskal–Wallis tests, followed by Dunn’s multiple comparisons. * *p* < 0.05, ** *p* < 0.01, *** *p* < 0.001, **** *p* < 0.0001. (**H**) Region selection of lung tissue obtained from COVID-19 patient using GeoMx WTA DSP with four-color staining (PanCK, CD68, CD45, DNA), modified from Delorey et al. [6]. (**I**) Region-specific expression profile for pro-inflammatory and pro-fibrotic mediators based on enrichment score derived from gene set enrichment analysis (GSEA) and expression for ADA2 representative from a single donor. (**J**) UMAP of scRNAseq profiles, colored according to ADA2 expression and to major cell categories. (**K**) Dotplot showing the average expression for selected genes across multiple cell types. ADA2^+^ cell types are highlighted in red.

The immunophenotypic analysis of circulating CD14^+^ monocytes showed a trending increase in classical CD14^+^CD16^−^ monocytes and a trending depletion of CD14^+^CD16^+^ monocytes in ARDS patients compared to those in the non-ARDS group (Figure 1A). Serum ADA2 activity, reflecting the circulating ADA2 protein levels, was significantly elevated in the ARDS patients compared to the non-ARDS patients (Figure 1B). Concurrently, flow cytometry showed reduced intracellular ADA2 protein levels in the circulating monocytes from the ARDS patients, as measured by mean fluorescence intensity (MFI) (Figure 1C). Notably, the serum ADA2 activity showed a significant correlation with the C-X-C motif chemokine 10 (CXCL10) levels (Figure 1D). Further analysis revealed that CXCR3, the receptor for CXCL10, had the highest frequency and expression among the chemokine receptors of ADA2^+^ leukocytes, when compared to CCR2 and CCR5 (Figure 1E,F). Additionally, serum ADA2 activity was found to be associated with arterial blood hypoxemia (Figure 1G), a key clinical feature of ARDS.

To further elucidate the role of ADA2, we analyzed the previously published spatial atlas and single-cell RNA sequencing (scRNAseq) data from COVID-19 lung tissue [6]. First, we analyzed data from a recently published spatial atlas of COVID-19 lung tissue [6]. Using Nanostring GeoMx Digital Spatial Profiling, regions of interest (ROIs) were categorized into inflamed and normal-appearing alveolar areas based on SARS-CoV-2 RNA hybridization signals (Figure 1H). Gene set enrichment analysis (GSEA) revealed an increase in the expression of pro-inflammatory and pro-fibrotic mediators in inflamed alveoli, when compared to normal-appearing alveoli. Notably, the expression of ADA2 was also elevated in inflamed alveoli, when compared to normal-appearing alveoli (Figure 1I). We further determined ADA2^+^ cells in COVID-19 lungs using a published scRNAseq dataset [6]. Our analysis confirmed a strong expression profile of ADA2 in myeloid cells (Figure 1J). Further analyses revealed that among the myeloid cell population, inflammatory CD14^high^/CD16^high^ monocytes exhibited the highest expression of ADA2 in COVID-19 lungs. These monocytes were characterized by an upregulation of interferon-inducing genes (*MX1, GBP2, IFIT3, OAS3, STAT1, IFIT1, IF144*) and of profibrotic genes, such as *SPP1* and *TREM2* (Figure 1K).

## 3. Discussion

The present study highlights key pathophysiological features of ARDS, linking hypoxia, infiltrative CXCR3 monocytes, and a monocyte-derived exoenzyme, ADA2. We demonstrate a significant association between infiltrative-monocyte-derived ADA2 and ARDS development, suggesting its role in driving lung inflammation and fibrosis.

Previous studies have shown a positive correlation between ADA2 activity and myeloid-cell-mediated diseases such as tuberculosis and macrophage activation syndrome [7]. Here, we provide evidence that monocyte-derived ADA2 is associated with ARDS. Serum ADA2 activity, reflecting extracellular ADA2 protein levels, was significantly elevated in ARDS patients and correlated with blood hypoxia. Meanwhile, the intracellular ADA2 levels in circulating monocytes decreased, suggesting the active secretion of ADA2 into the extracellular space under hypoxic conditions. Several studies have described a significant association between hypoxia and purinergic signaling. Eltzschig et al. found increased ADA activity in the plasma derived from patients with hypoxia, when compared to non-hypoxic control patients [8].

ADA2 is an enzyme secreted by activated monocytes and macrophages, which converts extracellular adenosine into inosine [3]. Elevated levels of inosine have been found in the plasma obtained from patients with severe COVID-19 disease, when compared to healthy controls or to non-severe patients by two different groups [9,10]. Consistent with our findings, these works suggest an important role of ADA2 signaling in modulating the hypoxic and severe condition of COVID-19-associated ARDS.

It is well established that enzymes responsible for converting ATP and AMP into adenosine, such as ENTPD1 (CD39) and CD73, are upregulated during hypoxia via *SP1* and *HIF1A* [4]. However, further studies are needed to understand the role of hypoxia in modulating ADA2 signaling in monocytes.

Infiltrative monocytes are known to drive pro-fibrotic responses in SARS-CoV-2-induced ARDS [1]. Here, we showed that CXCR3 is the most highly expressed chemokine receptor among ADA2^+^ leukocytes, and CXCL10—its ligand—is significantly associated with ADA2 activity in the serum of ARDS patients. Previous work has shown that mice deficient in CXCL10 or CXCR3 have reduced lung injury and improved survival in response to influenza virus infection [11].

Our prior research demonstrated that ADA2 was released into the extracellular space during macrophage differentiation, promoting pro-inflammatory and pro-fibrotic macrophage reprogramming through autocrine and paracrine mechanisms [3]. Previous scRNAseq data showed that ADA2 expression was upregulated in inflammatory CD14^high^/CD16^high^ monocytes in COVID-19 lungs. This cell population also expressed pro-fibrotic markers, such as *SPP1* and *TREM2* [6]. A spatial atlas of COVID-19 lungs showed that ADA2 expression was increased in areas of inflamed alveoli as compared to normal-appearing alveoli [6]. Together, these observations suggest an important link between ADA2^+^ circulating monocytes and a pro-inflammatory and pro-fibrotic phenotype in virus-induced lung injury.

In conclusion, our study suggests a significant association between myeloid-cell-specific ADA2 expression and ARDS development. However, a key limitation of the present study is the dichotomous analysis of blood- and lung-derived monocytes/macrophages in two different cohorts. Another limitation to this study is the impact of blood sampling processes for research purposes due to the nature of emergency scene medical care, which was impaired in some cases and might have led to selection bias.

Additionally, the absence of ADA2 in rodent models hinders mechanistic studies. Further studies using human tissue models are needed to understand the impact of ADA2 in modulating pro-fibrotic responses.

Despite these challenges, investigating the interplay between infiltrative CXCR3^+^ monocytes and ADA2 represents a promising therapeutic avenue for ARDS of viral origin.

## 4. Materials and Methods

### 4.1. Patient Cohort

Peripheral blood samples of patients with COVID-19 disease were collected at the Department of Gastroenterology, Infectiology, and Toxicology at University Hospital Heidelberg from March 2020 to January 2021. All patients provided written informed consent prior to inclusion in the study. The study received approval from the Ethics Committee of the University Hospital of Heidelberg, Germany (S148/2020). Patient care and research were conducted in accordance with the Declaration of Helsinki.

### 4.2. ADA2 Serum Activity, CXCL10 Serum Level, and Flow Cytometry

Serum samples were collected from each subject and were initially stored at −80 °C until the experiment was performed. Serum ADA2 activity was measured using a commercially available kit (Adenosine Deaminase Kit, DZ117A-K, Diazyme, Poway, CA, USA) and in the presence of a specific ADA1 inhibitor, erythro-9-(2-hydroxy-3-nonyl) adenine (EHNA), as previously described [3]. Peripheral blood mononuclear cells were isolated from whole blood by density gradient centrifugation on Ficoll–Paque (GE Healthcare, Sigma, Taufkirschen, Germany). Flow cytometry of the peripheral blood mononuclear cells were conducted, as mentioned in our previous work [3]. The following antibodies were used in this study: Anti-human CD14 PE (M5E2, Biolegend, San Diego, CA, USA); Anti-human CD16 APC (3G8, Biolegend); anti-human ADA2 (ab288296, abcam, Cambridge, UK); anti-rabbit AlexaFluor488 (Biolegend); anti-human CCR2 PE (K036C2, Biolegend); anti-human CCR5 APC (J418F1); and anti-human CXCR3 PE-Cy7 (G025H7, Biolegend). Cells were acquired using a BD LSR II Flow Cytometer (San Jose, CA, USA). Data were analyzed using the FlowJo v10 software.

The serum level of CXCL10 was measured, among others, by using the LEGENDplex^TM^ COVID-19 Cytokine Storm Panel (Biolegend, Catalog No. 741089).

### 4.3. Single Cell RNAseq and Digital Spatial Transcriptomics

Data were processed from our previous publication [6]. Briefly, a COVID-19 autopsy biobank of 17 donors obtained from 4 medical centers from northeastern United States during the first wave of the pandemic was used to generate a single-cell atlas of different organs. The clinical data and research specimens were collected in accordance with the institutional review boards of each center (see Delorey et al. [6]). The detailed description of sample acquisition and processing can be found from a previous study by Delorey et al. [6]. Computational methods are described in detail by Delorey et al. and in our recently published manuscript [6,12]. For the lung spatial atlas, a subset of donors was used. In brief, slides from formalin-fixed, paraffin-embedded blocks were stained against Pan-Cytokeratin, CD68, CD45, and DNA and the Whole Transcriptome Atlas (WTA) probe library (NanoString Technologies Inc., Seattle, WA, USA) to prepare for digital spatial profiling, according to the manufacturer instructions and, subsequently, loaded on the NanoString GeoMx DSP to obtain 20X fluorescent images. Sequencing reads of the region of interest were created as FASTQ files, which were then converted into Digital Count Conversion (DCC) files with NanoString’s GeoMx NGS DnD Pipeline and, subsequently, into an expression count matrix. A detailed description of the computational methods can be found in our previously published works [6,12]. The processed sequencing data and NanoString GeoMx raw and normalized count matrices are available on the Single Cell Portal (https://singlecell.broadinstitute.org/single_cell/study/SCP1052/; accessed on 5 December 2024) and on Gene Expression Omnibus under accession no. GSE163530.

Differential expression analysis from the scRNAseq dataset was carried out using the limma-trend method to detect cluster gene markers. First, genes expressed in at least 5% of the cells of at least one cluster were selected, and then UMI counts were normalized using the TMM normalization implemented in edgeR v.3.28.1. Then, a linear model “~Cluster + Center” was fitted to help model the mean–variance relationship with the limma-trend method and a robust empirical Bayes procedure. We used contrasts to compare the mean of a given cluster with all the others; a gene is considered a cluster marker if the contrast is significant at an FDR < 0.05, and if the cluster coefficient is higher than at least 75% of all other clusters.

### 4.4. Statistical Analysis

Statistical analysis was performed, and figures were created using GraphPad Prism 10.4 and using R Version 4.4.3, as previously described [3,6].

## Figures and Tables

**Table 1:** Basic characteristics of 34 COVID-19 patients requiring mechanical ventilation (ARDS group) and 33 non-critically ill COVID-19 patients without or receiving low-flow oxygen therapy (non-ARDS). *p* value was obtained using chi-squared statistics. NS = not significant. * *p* < 0.05, ** *p* < 0.01, *** *p* < 0.001, **** *p* < 0.0001.

Characteristics			
	ARDS (N = 34)	non-ARDS (N = 33)	*p*-value
Age (years, mean ± SD)	68 ± 12	59 ± 16	NS
Female gender (n, %)	9 (26%)	9 (27%)	NS
Oxygen therapy			
Total	34 (100%)	23 (70%)	*** *p* < 0.001
Mechanical ventilation (n, %)	34 (100%)	0 (0%)	
Low-flow oxygen therapy (n, %)	0 (0%)	23 (70%)	
Horowitz Quotient (mmHg, mean ± SD)	178 ± 44	>300	
PaO_2_ (mmHg, mean ± SD)	91 ± 14		
FiO_2_ (mean ± SD)	0.54 ± 0.14		
Main Comorbidities (n,%)			
Cardiovascular Disease	25 (74%)	12 (9%)	** *p* < 0.01
Hypertension	22 (65%)	11 (33%)	* *p* < 0.05
Diabetes	11 (24%)	2 (6%)	** *p* < 0.01
Pulmonary Disease	2 (6%)	1 (3%)	NS
Malignancy	7 (21%)	1 (3%)	* *p* < 0.05
Outcome (n)			
Death	14	0	**** *p* < 0.0001
Recovery	20	33	

## Data Availability

Raw data supporting the findings of this study are available in the controlled access repository DUOS (https://duos.broadinstitute.org/, accessed on 5 December 2024), under Dataset IDs DUOS-000126, DUOS-000127, DUOS-000128 and DUOS-000129. Processed scRNAseq and spatial atlas data from COVID-19 lung tissues are derived from the previous publication [6] and are available in the Gene Expression Omnibus (GEO, https://www.ncbi.nlm.nih.gov/geo/, accessed on 5 December 2024) under accession no. GSE171668 as well as no. GSE163530.
